# PIWI-interacting RNAs and PIWI proteins in glioma: molecular pathogenesis and role as biomarkers

**DOI:** 10.1186/s12964-020-00657-z

**Published:** 2020-10-27

**Authors:** Omid Reza Tamtaji, Mohammad Behnam, Mohammad Ali Pourattar, Michael R. Hamblin, Maryam Mahjoubin-Tehran, Hamed Mirzaei, Zatollah Asemi

**Affiliations:** 1 Research Center for Biochemistry and Nutrition in Metabolic Diseases, Institute for Basic Sciences, Kashan University of Medical Sciences, Kashan, Iran; 2 Halal Research Center of IRI, FDA, Tehran, Iran; 3 Department of Radiobiology, Iran University of Medical Sciences, Tehran, Iran; 4 Laser Research Centre, Faculty of Health Science, University of Johannesburg, Doornfontein, 2028 South Africa; 5 Student Research Committee, Mashhad University of Medical Sciences, Mashhad, Iran; 6 Department of Medical Biotechnology, Faculty of Medicine, Mashhad University of Medical Sciences, Mashhad, Iran

**Keywords:** piRNAs, PIWI protein, Glioma, Apoptosis, Migration, Invasion

## Abstract

Glioma is the most common primary brain tumor, and is a major health problem throughout the world. Today, researchers have discovered many risk factors that are associated with the initiation and progression of gliomas. Studies have shown that PIWI-interacting RNAs (piRNAs) and PIWI proteins are involved in tumorigenesis by epigenetic mechanisms. Hence, it seems that piRNAs and PIWI proteins may be potential prognostic, diagnostic or therapeutic biomarkers in the treatment of glioma. Previous studies have demonstrated a relationship between piRNAs and PIWI proteins and some of the molecular and cellular pathways in glioma. Here, we summarize recent evidence and evaluate the molecular mechanisms by which piRNAs and PIWI proteins are involved in glioma.

**Video abstract**

**Video abstract**

## Background

Glioma is known as the most common primary brain tumors and is one of the major global health problems worldwide. Glioma tumors can occur in different parts of the central nervous system (CNS) [1]. The World Health Organization (WHO) has classified gliomas into low-grade and high-grade; 10% are low-grade glioma (LGG) while 90% are high-grade glioma (HGG) [2]. Primary brain tumors are classified into four grades (I, II, III, and IV) based on their microscopic appearance, and their prognosis and therapy depend on the grade. LGGs are divided into grades I and II; including various types of astrocytoma, oligodendroglioma, gangliogliomas, desmoplastic infantile ganglioglioma, dysembroplastic neuroepithelial tumors and mixed glioma [3]. HGGs are divided into grades III and IV; grade III includes anaplastic astrocytoma, anaplastic ependymoma and anaplastic oligodendroglioma; grade IV includes gliosarcoma and glioblastoma multiforme (GBM) [4, 5]. Hypo-fractionated stereotactic radiotherapy is an effective treatment that helps to improve the quality of life in HGG [6]. In addition, a significant prolongation of survival is obtained by chemotherapy with a 15% relative reduction of the risk of death [7]. A study reported that temozolomide (TMZ) chemotherapy can be a valid option for treatment of LGG [8]. In addition, it has been reported that there was no significant difference between the effects of radiotherapy alone versus temozolomide chemotherapy alone in the treatment of patients with LGG [9].

Recently, it has been demonstrated that non-coding RNAs (ncRNAs) play essential roles in the pathophysiology and treatment of glioma. One of these types of small non-coding RNAs is Piwi-interacting RNAs (piRNAs) that are involved in the pathogenesis of glioma [3]. It has been shown that expression of a Piwi-like 1 protein was associated with Ki67 expression in gliomas [10]. Furthermore, piR-30,188 and PIWIL3 expression are decreased and negatively correlated with pathological grade of the glioma. piR-30,188 suppresses tumor cell proliferation, invasion, and migration, and promotes apoptosis [11]. PiR-8041 was also down-regulated (10.3-fold) in GBM relative to normal tissue, and acts to reduce cell proliferation [12].

piRNAs are formed from long intergenic transcripts, 3 UTRs of protein-coding RNAs, and ncRNAs [13]. Two major mechanisms are involved in the biogenesis of piRNAs, primary and secondary biogenesis [14]. The transcriptional processing of piRNAs includes the generation of pre-piRNAs, the modification of the 5 ′ and 3′ ends, and finally methylation. The pre-piRNA is produced by the movement of RNA polymerase in the 3 ′ to 5 ′ direction along the heterochromatin DNA strand [15]. The pre-piRNAs are transported out of the nucleus, and then bind to Yb bodies which are located around the mitochondria. Yb bodies are cytoplamisc organelles that were first discovered in Drosophila, and allow the PIWI proteins and piRNAs to assemble into a complex. The PIWI protein binds to the pre-piRNA to form piRISC by recognizing the 5 ′-end of piRNA [16]. The piRNAs play essential functional roles in epigenetic reprogramming, and can regulate transcription, translation, development and mRNA stability [17, 18]. The piRNAs can regulate transposable elements, probably through de novo DNA methylation [19]. In addition, piRNAs directly regulate chromatin architecture for control of genomic stability [17]. Target gene suppression by piRNAs is involved in transcriptional gene silencing (TGS), as well as post-transcriptional gene silencing (PTGS) in mice and flies [20]. It has been reported that the piRNAs are present in the CNS [21]. Rajasethupathy et al. [22] reported that there were 300 separate genomic regions that encoded piRNAs in the neurons of Aplysia (sea slugs). Another study reported that piRNA pathway genes were pivotal for multi-generational epigenetic memory in the *Caenorhabditis elegans* germline [23]. The piRNAs play an important role in the pathogenesis of brain-related disorders. piRNAs are abundant in the human brain, and may be biomarkers for the risk of Alzheimer’s disease [24]. Another study reported that tau-induced depletion of piRNAs led to neuronal mortality via transposable element dysregulation in tau-related neuro-degenerative disease [25].

PIWI proteins belong to the family of Argonaute proteins, which are abundantly expressed in animal and human germlines, where they contribute to gametogenesis and stem cell self-renewal [26]. It has been reported that the Piwi-like proteins can serve as clinical biomarkers and can detect cancers with poor prognosis [27, 28]. PIWI proteins are associated with several properties of tumor cells, including invasion, rapid growth, and apoptosis [29–31]. One study reported that the PIWI-piRNA complex was associated with expression of the neurotransmitter serotonin through CpG methylation of the cAMP-responsive element-binding protein 2 (CREB2) promoter [32]. The Piwi/piRNA complex is regulated by CREB2 in the Aplysia brain [22]. The PIWI-piRNA pathway is also involved in hepatocarcinogenesis [32]. In addition it was reported that PIWI proteins contributed to the pathogenesis of glioma [33]. Recently, the contribution of piRNAs and PIWI proteins to glioma has become an interesting topic for researchers, and a few studies have already been performed. These pathways might have a crucial role in the pathogenesis of many cancers including glioma.

Hence, it appears likely that these non-coding RNAs could be used as diagnostic, prognostic and therapeutic biomarkers in the treatment of glioma. Not only have deregulated piRNAs been detected in many cancer tissues, but the involvement of piRNAs in carcinogenesis and metastasis of several types of cancers has also been demonstrated. Moreover, the presence of piRNAs in human body fluids, (such as serum and plasma) make this class of non-coding RNAs more useful as biomarkers biomarkers [34]. In the same way as miRNAs, piRNAs are stable in plasma and blood for some time [35]. The most troubling problem confronting the use piRNAs as biomarkers is their detection methods, which are not as easy as miRNAs [36]. Moreover, each biological species has many unique piRNA sequences, and these sequences are not conserved between species. The sequence complexity of piRNAs makes it challenging to arrive at broad functional conclusions [17]. In recent years the role of miRNAs as biomarkers has been well explored, while piRNAs as the largest class of small non-coding RNAs expressed in animal cells, are beginning to be explored as biomarkers. However, more extensive studies are still needed to translate this finding into clinical applications [37].

Besides the use of non-coding RNAs (and piRNAs in particular) as prognostic and diagnostic biomarkers, these molecules can also be used as therapeutic targets. A few studies assessed the therapeutic roles of piRNAs in animal models of cancer. Tan et al., showed that the level of piRNA-36,712 was considerably lower in breast cancer compared to healthy breast tissue, and that it could indicate a poor clinical outcome in affected individuals [38]. Functional investigations showed that piRNA-36,712 could interact with RNAs generated by SEPW1P, a SEPW1 retro-processed pseudogene, and suppressed the expression of SEPW1 via competing with *SEPW1P* RNA for binding to miR-7 and miR-324. Moreover, it was shown that the increased expression of SEPW1 following piRNA-36,712 down-regulation could inhibit P53 in breast cancer, resulting in decreased levels of E-cadherin and P21, and up-regulated levels of Slug, thereby increasing the proliferation, migration, and invasion of the cancer cells. Additionally, they found that piRNA-36,712 exerted a synergistic antitumor effect when combined with doxorubicin or paclitaxel, as chemotherapeutic drugs [38].

In the present review we summarize recent supporting evidence and evaluate the mechanisms by which piRNAs and PIWI proteins could be involved in glioma.

### Biogenesis of piRNAs

**Fig. 1:**
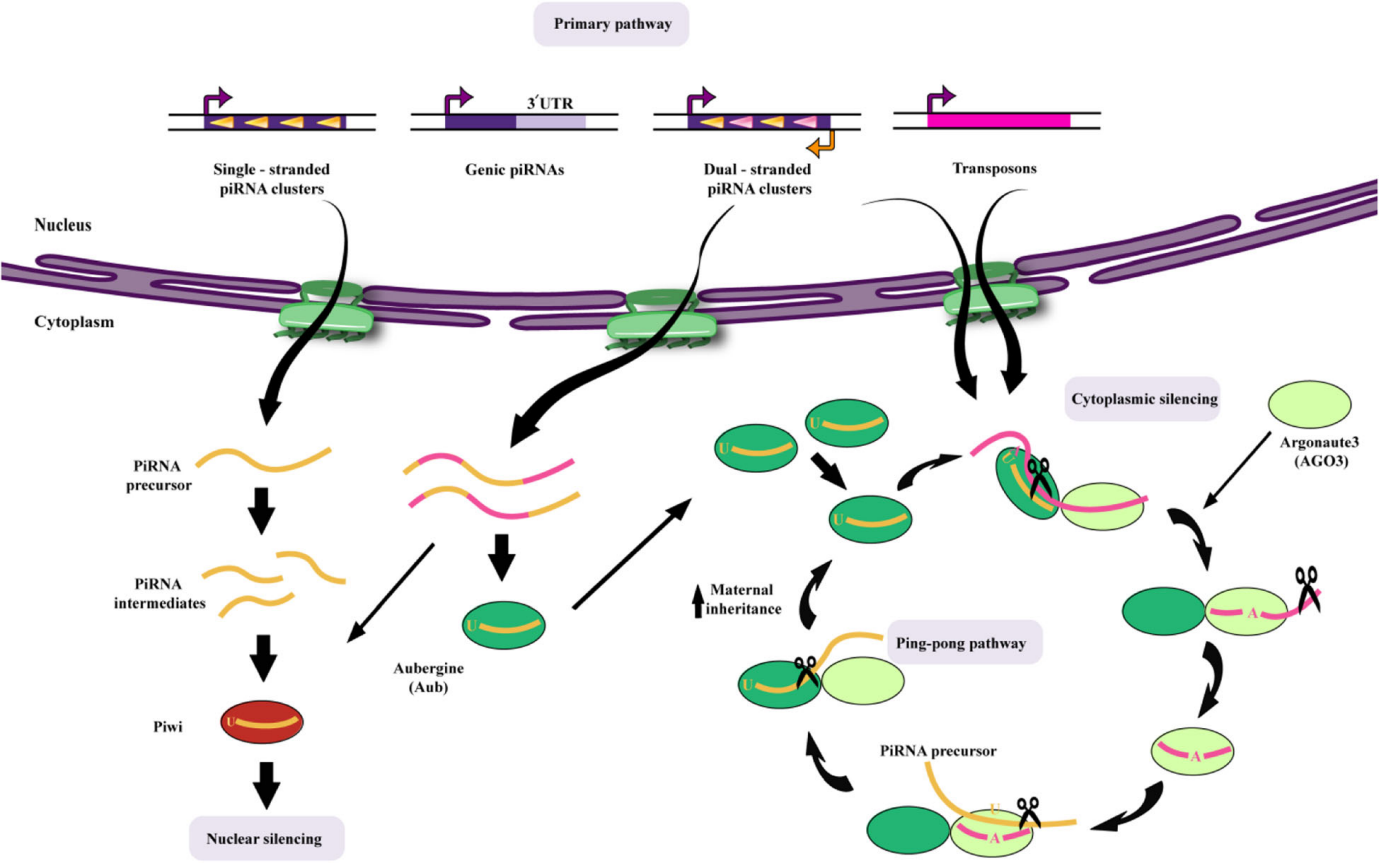
Biogenesis pathway of Drosophila piRNAs, showing the primary and ping-pong pathways. In the primary pathway, piRNAs are transcribed from genomic regions called piRNA clusters, processed, and loaded onto Piwi or Aub. The 3′-UTR sequences of some protein-coding genes can also serve as a source of primary piRNAs. Gene silencing takes place both in the cytoplasm and the nucleus (also see Fig.3). Piwi performs transcriptional gene silencing in the nucleus. Together with AGO3, the Aub–piRNA complex serves as a trigger to start the ping-pong amplification pathway. The ping-pong pathway silences the expression of the target transposon sequence and amplifies the piRNA sequence at the same time. Note that some Aub–piRNA complexes are also maternally inherited. Abbreviations: piRNA,PIWI-interacting RNA; UTR, untranslated region

**Fig. 2:**
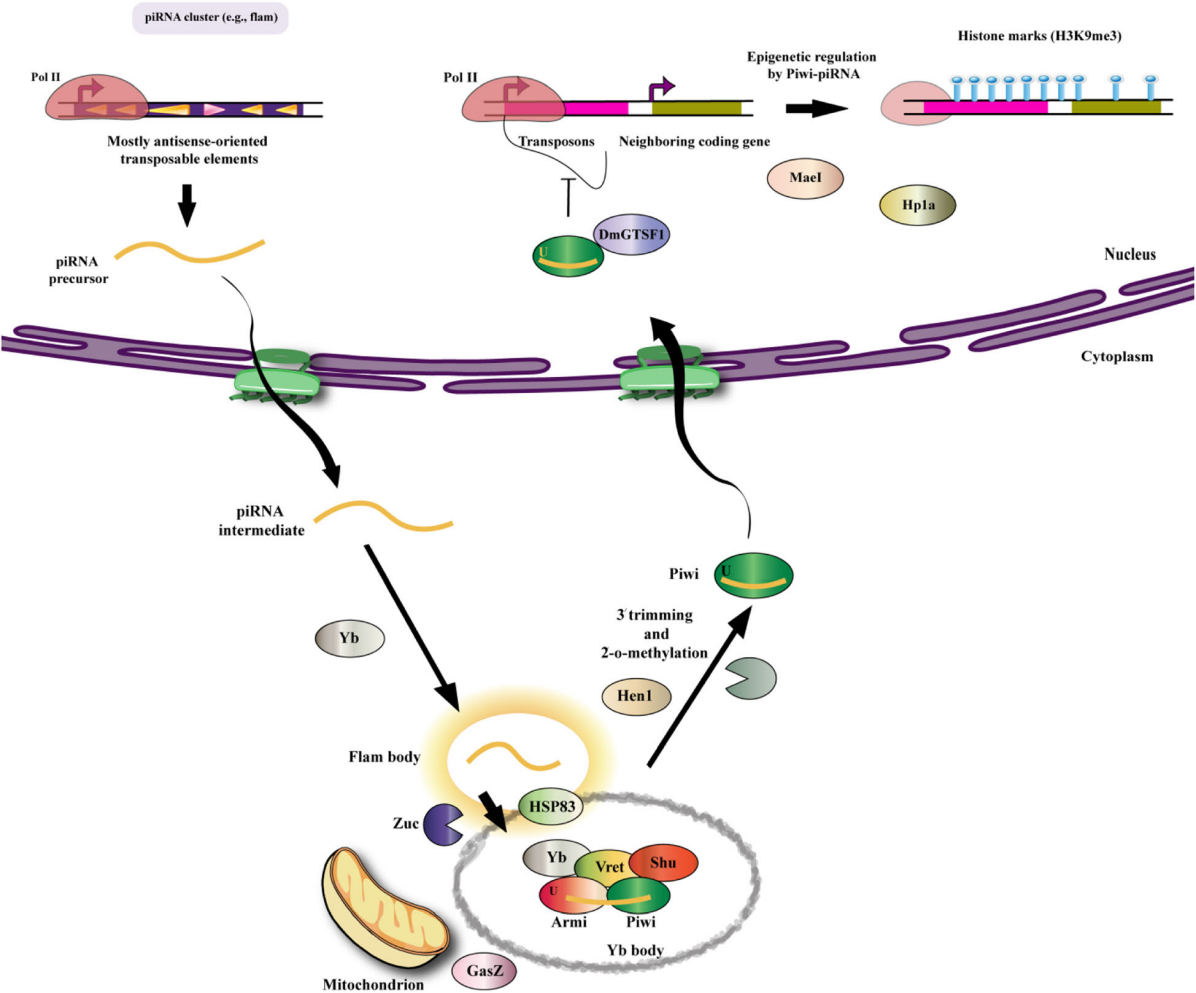
Epigenetic silencing by Piwi–piRNA in Drosophila. **a** Piwi, a Drosophila PIWI protein, is localized to the nucleus and can epigenetically silence target genes. Transcripts of piRNA clusters, which contain numerous sequences complementary to transposons, serve as precursors to piRNAs. piRNA precursors are processed into piRNA intermediates and exported to the cytoplasm. Intermediates are processed by the endonuclease Zuc near the mitochondria, localized to granules termed Flam bodies, and then to Yb bodies, where factors such as Yb, Armi, Vret, and Shut are localized. Armi is recruited to mitochondria by Gasz. Here, piRNAs are processed and loaded onto Piwi. Then, piRNAs are 3′trimmed and 2′-O-methylated by Hen1 and then transferred into the nucleus. Within the nucleus, Piwi–piRNA complexes regulate their target genes by modifying histones and affecting the association of Pol II with target genes. Several factors, such as DmGTSF1, Mael, and HP1a, are involved in this process, but the regulatory mechanism remains to be completely understood. Abbreviations: Armi, Armitage; Mael, Maelstrom; Mito, mitochondria; N, nucleus; piRNA, PIWI-interacting RNA; Pol II, RNA polymerase II; Shut, Shutdown; TE, transposable element; Vret, Vreteno; Yb, fs (1) Yb; Zuc, Zucchini. Scale bar, 0.2 μm

There are two different pathways for the production of piRNAs, called the “primary processing pathway” and the “ping-pong cycle” or secondary pathway (Fig.1). In this respect, primary piRNAs contain uridine (U) at their 5′ nucleic acid, whereas secondary piRNAs show a 10-nt complementary binding sequence with primary piRNAs at their 5′ end and are biased with adenosine bases [5, 21, 22, 29, 31]. The primary pathway operates in germline cells as well as somatic cells in Drosophila ovaries, while the ping-pong cycle only functions in germline cells. The Piwi protein is the only one of the PIWI sub-family members in Drosophila ovarian somatic cells, where the piRNA precursors are encoded by piRNA clusters e.g. the flamenco (flam) locus and processed by successive steps **(**Fig.2**)** [21]. In this regard, the flam locus contains many truncated transposons that are mostly antisense-oriented, compared with the coding strands of the transposons [21, 41, 42]. The main transcripts from the piRNA clusters are then transferred to the cytoplasm and processed into intermediates, albeit these processes are not completely understood. The endonuclease Zucchini (Zuc) is situated on the outside of mitochondria and has been assumed to be vital for preparing precursor RNAs [43, 46], but whether RNAs with U at the 5′ end can be produced by Zuc should be investigated. The loading and development steps most likely take place in peri-nuclear granules identified as Yb bodies and located on the outside of the mitochondria (Fig.2). Moreover, components of the the Yb bodies, such as fs (1) Yb (Yb), Vreteno (Vret), Armitage (Armi), Shutdown, and Sister of Yb (SoYb) are required for producing the final piRNAs [47, 52]. Furthermore, GasZ and Minotaur (Mino) are restricted to mitochondria and also participate in primary piRNA production [53, 54]. Although the association between mitochondria and piRNA biogenesis is accepted, it is unclear exactly which mitochondrial functions are involved. piRNA precursors are transformed into the final piRNA sizes via an obscure 3′-5′exonuclease activity [55]. First the the DmHen1/Pimet piRNA methyl-transferase adds a 2′-O-methyl group to the 3′ ends of piRNAs in order to allow the formation of the Piwi-piRNA complex and Piwi-piRISCs [56, 57]. The Piwi-piRNA complex is transported to the nucleus in order to control the transcription of target genes [58]. piRNA-free Piwi also remains in the cytoplasm, implying that the Yb body is the location to assemble the functional piRISCs. It should be noted that only the functional complex can be transported into the nucleus [47, 48]. Transcripts or their intermediates originating from the flam locus, also accumulate at peri-nuclear locations adjacent to the Yb bodies, known as flam bodies [59], whose formation is controlled by the RNA-binding activity of Yb. This suggests that Yb incorporates primary piRNA transcripts and related intermediates into the flam body. Primary piRNAs are produced from double-stranded piRNA clusters, including the 42AB locus in Drosophila germline cells, and are then loaded into Aub and Piwi to assemble the piRISCs. Moreover, germline piRNAs are lower in Drosophila mutants with reduced expression of Armi [60], implying that somatic components are also needed for the biogenesis of germline piRNAs. Nevertheless, the existence of germline equivalents to the components of Yb bodies should be recognized. Therefore, the primary piRNA pathway is somewhat different between germline cells and somatic cells, but how the primary pathway functions in Drosophila germline cells is not completely clear. Moreover, mammalian orthologs related to the factors that govern somatic primary piRNA biogenesis in Drosophila have been discovered [61, 65]. As one example, mitoPLD is a mouse ortholog of Zuc, identified as a mitochondrial protein implicated in piRNA generation [63, 65]. Furthermore a mouse ortholog of Armi (MOV10L1) is an RNA helicase, which has been proposed to be involved in the biogenesis of piRNA [62, 66]. Therefore mammalian primary piRNAs are likely to be generated by means of pathways resembling those of Drosophila, although further experimental studies are needed to confirm this hypothesis.

### Distribution and physiological functions of Piwi and piRNAs

Two different approaches have been employed for the detection of piRNAs. The first approach utilizes sequence-based features to identify the piRNAs. piRNAs tend to have uridine at the 5′ cleavage sites, and can be identified as piRNAs by checking the occurrence of uridine within the 10 upstream and downstream bases [39]. The prediction accuracy of this approach is between 61 and 72% for mouse piRNAs. The analysis of K-mer sequences provides the spectrum of K-mers (*k* = 1–5). All 1364 K-mers from 1-mer sequences to 5-mer sequences were analyzed to predict the occurrence of piRNAs [40]. The second approach uses bioinformatics analysis of the clustering locus within genomic piRNA clusters for piRNA detection [41]. Currently the gold standard methods for detection of piRNA expression are Northern Blot analysis and in situ hybridization, although a multiplex detection method based on real-time PCR for profiling of multiple piRNAs has been developed [42].

Several studies have examined the distribution of piRNAs within the brain of experimental animals. For instance, in multiple regions of the mouse brain, including the hippocampus, Piwi mRNA expression was detected by in situ hybridization. Lee and colleagues reported that there were plentiful piRNA detected in mouse dendritic spines, and that knockdown of piRNAs led to decreased spine density in the axons [21]. piRNAs were shown to be present in *Aplysia* brain neurons [22], and their mutations affected the neuron function as well as brain development. Further investigations also demonstrated the potential roles of piRNA in the brains of many different organisms [43, 44]. Lee and co-workers sequenced small RNA libraries obtained from the mouse hippocampus to identify small non-coding RNAs (ncRNAs) (≤35 bp) using RNA-Seq technology (30×) [21]. This study generated a total of 9.18 × 10^6^ reads in the female mouse brain and 14.83 × 10^6^ 35-bp reads in the male mouse brain. Among these reads, 66.7% mapped to the mouse genome, accounting for 9.89 × 10^6^ (male) and 6.12 × 10^6^ (female) unique RNA transcripts. After filtering out small RNAs < 25 nt, miRNAs, adaptors, rRNAs, and tRNAs, 11.3% of these transcripts ranged from 25 to 32 nt. Among these piRNA-like small RNAs with 25–32 nucleotides, 2297 (0.76%) were confirmed as piRNAs and deposited in the piRNA databank (pirnabank.ibab.ac.in) or the ncRNA database (RNAdb).

Serotonin is a neurotransmitter that is mainly produced within the serotonergic neurons of the CNS, and acts to modulate sleep, appetite, and mood. Its regulation of synaptic transmission contributes to the pharmacological effects of antidepressants drugs, and it also affects cognitive functions, including learning and memory. In the *Aplysia* brain, it was found that the levels of Piwi/piRNA complexes were sensitive to serotonin regulation [22]. Moreover, CREB2, a transcriptional repressor binds to the cAMP-responsive element (CRE), and has a role in development of the nervous system. CREB2 is an important memory suppressor gene in neurons that constrains the growth of new synaptic connections,. It has been shown that the Piwi/piRNA complex modulates the activity of CREB2 in the *Aplysia* brain [22]. In neurons, the methylation of the serotonin-dependent conserved CREB2 promoter CpG island is facilitated by Piwi/piRNA complexes, thereby modulating memory storage, learning-associated synaptic plasticity, and long-term enhancement of synaptic transmission [22]. Also, the Piwi/piRNA complex plays a role in synaptic transmission in mouse neuronal dendrites [21]. It is likely that Piwi/piRNA complexes regulate the development of dendritic spines [21]. One study revealed that Piwi/piRNA acts to carry out transcriptional and post-transcriptional silencing of the alcohol dehydrogenase gene (*Adh*) in Drosophila [44, 45]. *The main site of expression of Adh* is the liver, however, it is also expressed in the brain [46]. Its homolog, the *ADH* gene, is a major risk facor gene for alcohol dependence as reported by many genome-wide association studies (GWASs) and candidate gene investigations [47]. piRNA activity has been detected in various mammalian brain samples, but this may be different from humans because of the poor conservation across species. However, these studies have provided some clues about possible roles of Piwi/piRNAs in human brain diseases [48].

As discussed earlier, the main function of piRNAs is to regulate transposons. This raises the question of whether piRNAs play an important role in brain tumors, because transposition events are commonly seen in human brain cancer cells [48]. This may be supported by evidence indicating that inactivation of Aub or Piwi in Drosophila, inhibits the growth of lethal malignant brain tumors [49]. Another clue regarding piRNA function in the brain was provided by the discovery of L1 retrotransposons in rat, mouse, and human brain samples. L1 retrotransposons have been implicated in somatic mosaicism of neuronal cells, heterogeneity, and differentiation in the brain [50, 51]. A number of retrotransposons and piRNAs co-exist within the brain. L1 retrotransposons are regulated by these piRNAs, and piRNA mutants show increased retrotransposon expression in the brain. The combination of retrotransposons and piRNAs probably plays an essential role in the brain and its development [48].

### piRNAs in gliomas

**Fig. 3:**
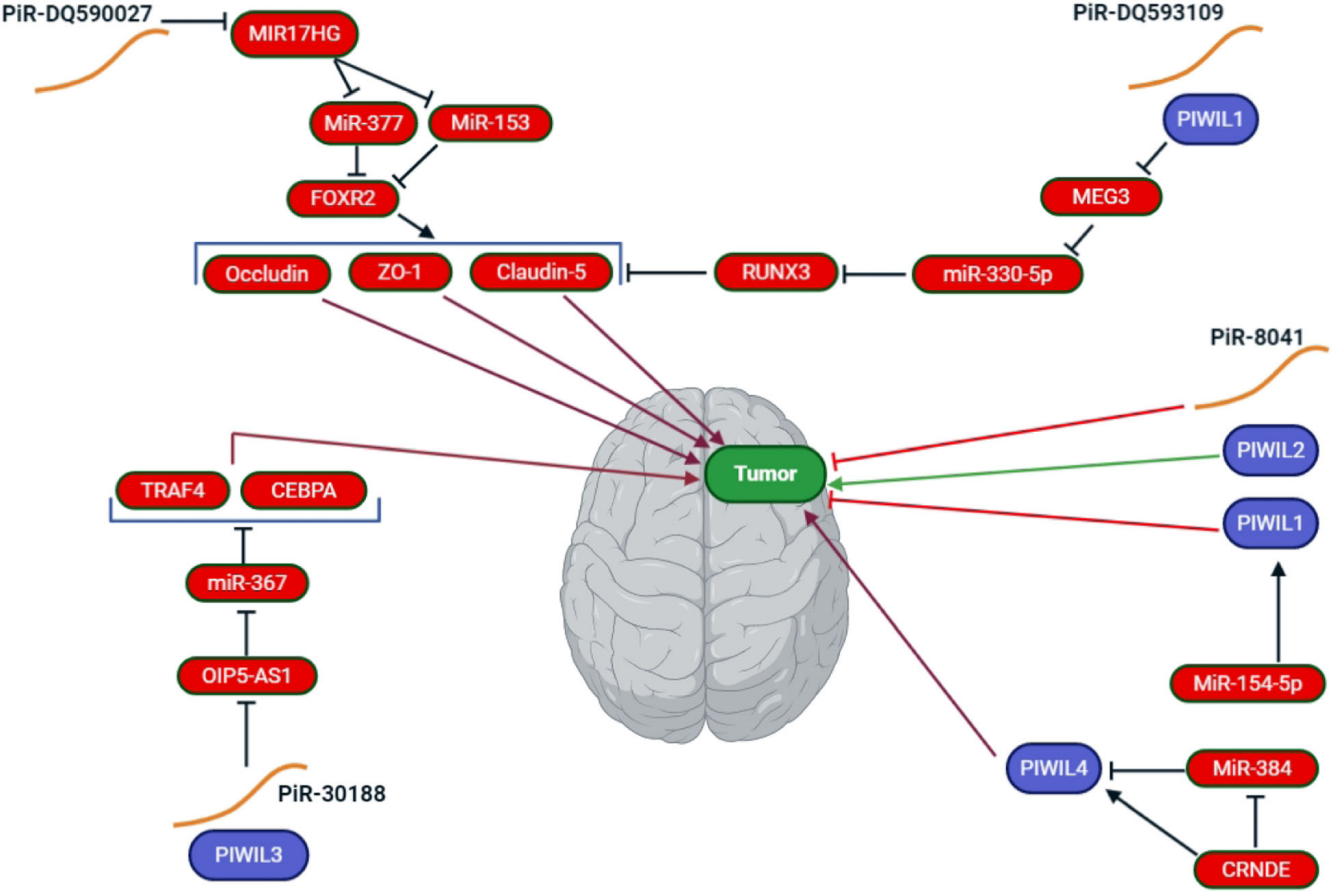
Schematic representation of the molecular signaling pathways targeted by piRNAs and PIWI proteins in glioma. CEBPA, CCAAT/enhancer binding protein alpha; MEG3, maternally expressed gene 3; RUNX3, Runt-related transcription factor 3; TRAF4, TNF receptor-associated factor 4, ZO-1, zonula occludens-1

Recently, it has been shown that piRNAs are involved in the tumorigenesis processes in several different organs by epigenetic mechanisms [52]. However, few studies have evaluated the role of piRNAs in gliomas (Table 1, Fig. 3). Jacobs et al. [3] reported that variant rs147061479 in piR-598 elevated the risk of glioma by using qPCR and genome-wide expression profiling. They found that rs147061479 abolished the tumor-suppressive function of piR-598, instead conferring tumor growth-promoting properties. In another study using array-based piRNA profiling as well as expression difference analysis of GBM relative to normal tissue, they found that the expression levels of piR-15,988, piR-20,249, piR-54,022 and piR-8041 were all reduced in GBM. Furthermore, these differences in expression were confirmed in individual samples using qPCR. Interestingly, piR-8041 was reported to be approximately 15-fold and 35-fold lower expressed in two GBM cell lines (U87 and A172) [12]. Pretreatment with piR-8041 remarkably decreased the rapid growth of glioma cells, caused cell cycle arrest and apoptosis, inhibited cell survival pathways, and reduced the tumor volume in vivo [12].

**Table 1:** Studies reporting the role of piRNAs and PIWI proteins in glioma

piRNA/PIWI proteins	Disease/samples	Expression	Publication Year	Ref
piR-8041	GBM/Human tissue	Down-regulated	2018	[12]
piR-15,988	GBM/Human tissue	Down-regulated	2018	[12]
piR-20,249	GBM/Human tissue	Down-regulated	2018	[12]
piR-54,022	GBM/Human tissue	Down-regulated	2018	[12]
piR-DQ590027	Glioma/Cell line	Down-regulated	2018	[59]
piR-DQ593109	Glioma/Cell line	Up-regulated	2018	[82]
piR-30,188	Glioma/Human tissue and cell line	Down-regulated	2018	[11]
PIWIL1	Glioma/ Cell line	Up-regulated	2018	[82]
	GBM/Human tissue and cell line	Up-regulated	2018	[33]
PIWIL2	Glioma/Human tissue and cell line	Up-regulated	2017	[67]
PIWIL3	Glioma/Human tissue and cell line	Down-regulated	2018	[11]
PIWIL4	Glioma/Human tissue and cell line	Up-regulated	2016	[68]
piR-598	Glioma/Human tissue and cell line	Down-regulated	2016	[3]
piR-18,913/ rs62435800	Glioma/Human tissue and cell line	Polymorphism	2016	[3]
piR-598/rs147061479	Glioma/Human tissue and cell line	Polymorphism	2016	[3]
piR-11,714/rs142742690	Glioma/Human tissue and cell line	Polymorphism	2016	[3]
piR-3266/rs35712968	Glioma/Human tissue and cell line	Polymorphism	2016	[3]
piR-2799/ rs149336947	Glioma/Human tissue and cell line	Polymorphism	2016	[3]

The microRNA miR-153 is also involved in pathogenesis of gliomas [53]. MiR-153 is a tumor suppressor gene that causes apoptosis and suppresses migration and invasion in glioma [54–56]. Moreover, miR-377 is also implicated in the pathogenesis of glioma, because it suppressed proliferation and invasion in glioma cells [57]. In addition, the transcription factor FoxR2 increased glioma proliferation and tumorigenicity [58].

It has been reported that piR-DQ590027 is reduced in glioma-derived endothelial cells (GECs). Up-regulation of piR-DQ590027 decreased the expression of occludin, claudin-5 and ZO-1, and increased the permeability of the blood-brain barrier (BBB) [59]. It is known that increased blood-tumor barrier permeability is associated with the down-regulation of occludin, ZO-1, and claudin-5 [60]. Furthermore, a key step in brain metastasis is the interaction and penetration of the BBB by cancer cells [61]. Therefore, up-regulation of piR-DQ590027 might be a mechanism for increasing the BBB permeability as well as penetration by cancer cells. Moreover, piR-598, piR-8041 and piR-DQ590027 may be used as diagnostic markers for glioma tumors.

Jacobs et al. carried out a study to evaluate the roles of piRNAs in glioma. They analyzed the genetic variants in 1428 separate piRNAs and their association with glioma risk, using imputed and measured genotypes from the GliomaScan genome-wide association study (2401 controls and 1840 cases) [3]. To investigate the functional effect of the most recognized piRNA and its variant allele, an in vitro assay was also conducted. Variants in five piRNAs were found to be associated with glioma riak, and four of these showed narrow clusters of higher association signals surrounding the index variant. piR-598 functional analysis showed that wild-type piRNA transfection affected the expression of genes implicated in cell death and survival, and attenuated colony formation and glioma cell viability. On the other hand, transfection with piR-598 containing the variant allele at rs147061479 increased cell proliferation. Use of genetic association analysis has identified numerous piRNAs related to glioma risk, and follow-up functional analysis suggested that variant rs147061479 in piR-598 increased glioma risk by abolishing the tumor-inhibitory property of piR-598, and instead conferring growth-promoting properties [3].

### PIWI proteins in glioma

PIWI proteins could be an appropriate target for cancer therapy [62]. The microRNA miR-154-5p is involved in the pathogenesis of different cancers, and has been shown to inhibit migration, invasion and proliferation in prostate cancer cell lines by targeting E2F5 [63]. PIWIL1 is a target for miR-154-5p, and this may explain the anti-cancer effects of miR-154-5p [33]. Another study reported that miR-154 could suppress migration and invasion in non-small cell lung cancer by regulating the epithelial-mesenchymal transition via targeting Zinc finger E-box binding homeobox 2 [64]. One study reported that miR-154 expression was down-regulated in glioblastoma tissues [65]. Another study reported that up-regulation of miR-154-5p inhibited rapid growth and metastasis of GBM, and promoted apoptosis, while inhibition miR-154-5p expression had the opposite effects. It was proposed that up-regulation of miR-154-5p exerted these changes via targeting PIWIL1 in glioblastoma [33].

The Ki-67 index measures proliferation of cells in human glioma and is correlated with the histological classification of tumors [66]. There has been found to be a correlation between PIWIL2 expression, and the Ki-67 index and the grade of human glioma. PIWIL2 may therefore be a prognostic factor for survival of glioma patients. In vitro studies showed that knock-down of PIWIL2 in glioma cells induced cell cycle arrest and promoted apoptosis. In addition, silencing of PIWIL2 expression inhibited the migration of glioma cells [67].

The miR-384/PIWIL4/STAT3 axis has an important role in pathogenesis of glioma [68]. The colorectal neoplasia differentially expressed (CRNDE), is a lncRNA with an important role in the growth and progression of different cancers [69]. The expression of CRNDE was shown to be significantly elevated in glioma tissue. The CRNDE over-expression was correlated with increased tumor size, higher grade and likelihood of recurrence. Moreover, up-regulation of CRNDE expression was also related to poor survival in glioma patients [70]. Increased expression of lncRNA CRNDE in human glioma was implicated in increased cell migration and proliferation [71]. CRNDE up-regulation promoted rapid cell growth, invasion and migration, while also suppressing apoptosis in glioma cells [72]. MiR-384 has been found to be down-regulated in glioma tissue, and in vitro it significantly suppressed proliferation, invasion, and migration of glioma cells [73]. Both CRNDE knock-down or miR-384 up-regulation led to a decrease in PIWIL4 in glioma. In addition, some down-stream proteins of PIWIL4, including STAT3, cyclin D1, SLUG, VEGFA, MMP-9, Bcl-2, Bcl-xL and caspase 3 were regulated by treatment with miR-384 and PIWIL4 [68].

In one study, Sun et al., evaluated the clinical significance of Hiwi (human equivalent of Piwi) in glioma. They found that Hiwi was specifically expressed in most glioma samples, and the levels correlated with higher tumor grades [74]. Statistically, it was determined that patients with high Hiwi expression had poorer outcomes compared to individuals with low expression of Hiwi. They concluded that Hiwi was an important factor in the progression of glioma, and could be a candidate as a biomarker for diagnosis and prognosis of malignant glioma [74].

### Interactions between PiRNAs and PIWI in glioma

Piwi proteins and piRNA transcripts are localized in the mitochondrial fractions of somatic cancer cells [75]. It is well-known that there is a relationship between epigenetic modifications (such as histone alterations and DNA hypo/hyper-methylation) and the development and progression of cancer [76]. piRNA/PIWI complexes may be involved in tumorigenesis via abnormal DNA methylation leading to genomic silencing and an increased stem-like state [52]. Furthermore, there is a complex interaction between piRNAs and miRNAs that can modulate cellular processes. It was found that the repression of a piRNA amplification loop by miR-17-5p led to increased levels of transposon mutagenesis. This happened because the amplification loop of piRNA had an identical 5′ sequence, and could target Mili/Miwi2 (an essential component of the piRNA amplification loop) as well as the DNA methyltransferase, Dnmt3a [37]. However, the exact mechanisms of interaction between piRNAs and miRNAs was not completely elucidated.

The lncRNA called maternally expressed gene 3 (MEG3) has an important role in the pathogenesis of different cancers by affecting cell proliferation and apoptosis [77, 78]. Moreover, MEG3 strongly decreased tumor growth and volume, and the expression of proliferating cell nuclear antigen (PCNA) and Ki67. MEG3 also suppressed miR-93 and inhibited the PI3K/AKT pathway in glioma [79]. Runt-associated transcription factor 3 (RUNX3) is another tumor suppressor gene, which has been demonstrated to show lower expression in human glioma [80, 81]. The PIWIL1/piRNA-DQ593109 (piR-DQ593109) is known to be a central regulator of blood-tumor barrier (BTB) permeability. PIWIL1 and piR-DQ593109 were over-expressed in glioma-derived GECs. Down-regulation of PIWIL1 and piR-DQ593109 promoted BTB permeability. Moreover, piR-DQ593109 and PIWIL1 lowered MEG3 expression, while restoration of MEG3 abrogated the post-transcriptional suppression of RUNX3 by sponging miR-330-5p. Therefore, down-regulation of PIWIL1 and piR-DQ593109 increased BTB permeability via affecting the MEG3/miR-330-5p/RUNX3 axis [82].

PIWIL3 plus piR-30,188 and the PIWIL3/OIP5-AS1/miR-367-3p/CEBPA (CCAAT/enhancer-binding protein alpha) complex is involved in the pathogenesis of glioma [11]. It has been shown that miR-367-3p has an important role in the pathogenesis of cancer [83, 84]. MiR-367 regulated cell metastasis and proliferation via targeting metastasis-associated protein 3 (MTA3) [85]. miR-367-3p improves the effects of Sorafenib chemotherapy by supressing pAKT and pERK signaling [86]. A study demonstrated that low expression of miR-367 was linked to progression and a poor clinical outcome in glioma patients [87]. One study reported that PIWIL3, piR-30,188 and miR-367-3p were decreased and OIP5-AS1 was increased in glioma. Up-regulation of miR-367-3p, piR-30,188 and PIWIL3 or knockdown of OIP5-AS1 led to suppression of glioma progression. PiR-30,188 was found to bind to PIWIL3, however up-regulation of piR-30,188 and PIWIL3, jointly or separately, suppressed OIP5-AS1 expression. Moreover, up-regulation of miR-367-3p also reduced OIP5-AS1. CEBPA and TRAF4 are both over-expressed in glioma cells and tissues, and show a positive correlation with the pathological grade of glioma. Increased expression of PIWIL3 and piR-30,188, or the reduction of OIP5-AS1, or their combined application suppressed TNF receptor-associated factor 4 (TRAF4) and CEBPA expression [11].

## Conclusions

Glioma is most common primary brain tumor, with high mortality throughout the world. Several risk factors have been determined for glioma that could help in its diagnosis, but timely diagnosis and prediction of treatment outcome are important issues for oncologists. Recently, piRNAs and PIWI proteins have been attracting much attention for diagnosis and prediction of different diseases. Researchers have identified different piRNAs and PIWI proteins which are expressed in glioma cells and tumors. These reports have indicated that piRNAs and PIWI proteins could be promising biomarkers for the diagnosis and prognosis of glioma. In addition, several studies have been performed to evaluate the roles of specific piRNAs and PIWI proteins in the pathogenesis of glioma. PiR-598, piR-8041, piR-DQ590027, piR-DQ593109, PIWIL1, PIWIL2, PIWIL3, and PIWIL4 may all be involved in the pathogenesis of glioma, and could be diagnostic markers for glioma. However, further studies are necessary to discover the mechanisms of action of piRNAs and PIWI proteins in glioma initiation and progression. Moreover, it has been proposed that a web-server should be set up to display their findings in a flexible way, so that users can manipulate the display and input their data as desired. Such a database would certainly be very useful for drug design.

### Future perspectives

PiRNAs have recently been found to be expressed across diverse cancer types in a tissue-specific manner. Deregulated piRNAs have been detected in colon, gastric, lung, breast, bladder, uterine, thyroid, and kidney cancer tissues. Abnormal expression of piRNAs is a signature finding with valuable prognostic or diagnostic implications for several types of cancer. Of note, it has been observed that piRNAs can be detected in human body fluids, such as the plasma and serum of healthy people as well as cancer sufferers, in a significantly stable form [34, 88]. Taken together these findings suggest that piRNAs could be used as diagnostic, prognostic or therapeutic biomarkers in the treatment of several cancers such as gliomas.

## Data Availability

Not applicable.

## Abbreviations

BBB: Blood-brain barrier
CNS: Central nervous system
CREB2: cAMP-responsive element-binding protein 2
CRNDE: Colorectal neoplasia differentially expressed
GWASs: Genome-wide association studies
HGG: High-grade glioma
LGG: Low-grade glioma
MEG3: Maternally expressed gene 3
MTA3: Metastasis-associated protein 3
PTGS: Posttranscriptional gene silencing
TGS: Transcriptional gene silencing
TMZ: Temozolomide
WHO: World Health Organization
